# Correction: Effects of tobacco smoke and electronic cigarette vapor exposure on the oral and gut microbiota in humans: a pilot study

**DOI:** 10.7717/peerj.4693/correction-1

**Published:** 2018-08-23

**Authors:** Christopher J. Stewart, Thomas A. Auchtung, Nadim J. Ajami, Kenia Velasquez, Daniel P. Smith, Richard De La Garza, Ramiro Salas, Joseph F. Petrosino

**Affiliations:** 1 Alkek Center for Metagenomics and Microbiome Research, Department of Molecular Virology and Microbiology, Baylor College of Medicine, Houston, TX, USA; 2 Institute of Cellular Medicine, Newcastle University, Newcastle, UK; 3 Menninger Department of Psychiatry and Behavioral Sciences, Baylor College of Medicine, Houston, TX, USA; 4 Veteran Affairs Medical Center, Michael E. DeBakey VA Medical Center, Houston, TX, USA; 5 Department of Neuroscience, Baylor College of Medicine, Houston, TX, USA

Corrigendum:

After publication, it was pointed out to the authors that some of the language implied causality. In order to avoid potential misinterpretation, they have edited the language by replacing “increased” with “higher”, replacing “decreased/reduced” with “lower”, replacing “changed/altered” with “different”, and removing the phrase “the effect(s) of” throughout the introduction, results, discussion, and conclusion.

They have also removed “From a microbial ecology perspective, this study supports the perception that electronic cigarettes represent a safer alternative to tobacco smoking” from the conclusion in order to avoid language that might be considered as providing medical advice. Similar language was also removed from the abstract’s discussion section.

In the discussion, the authors oversimplified a sentence relating to some species of *Bacteroides* considered probiotic and to avoid misrepresentation they have also added “though some [*Bacteroides*] are considered opportunistic human pathogens”.

The authors confirm that none of these changes affect the overall reported associations or the conclusions of the work.

