# Supplementary material for: Effects of tobacco smoke and electronic cigarette vapor exposure on the oral and gut microbiota in humans: a pilot study
Source: PeerJ. 2018 Apr 30;6:e4693. doi: 10.7717/peerj.4693 (PMC5933315; doi:10.7717/peerj.4693)
Supplement: Supplemental Information 1 [file peerj-06-4693-s001.docx]

**Table S1. Correlations between the top 10 most abundant genera per sample type and the CO levels based on linear regression**

| Taxa | Sample | R-Squared | P-Value | FDR-Adj. P |
| --- | --- | --- | --- | --- |
| *Bacteroides* | Feces | 0.229 | 0.009 | **0.043** |
| *Faecalibacterium* | Feces | 0.002 | 0.824 | 0.883 |
| *Prevotella_9* | Feces | 0.314 | 0.002 | **0.016** |
| *Eubacterium_rectale_group* | Feces | 0.050 | 0.246 | 0.410 |
| *Lachnospiraceae_UCG_008* | Feces | 0.079 | 0.139 | 0.410 |
| *Blautia* | Feces | 0.051 | 0.241 | 0.410 |
| *Subdoligranulum* | Feces | 0.057 | 0.213 | 0.410 |
| *Fusicatenibacter* | Feces | 0.003 | 0.773 | 0.883 |
| *Ruminococcus_2* | Feces | 0.001 | 0.883 | 0.883 |
| *Bifidobacterium* | Feces | 0.013 | 0.558 | 0.798 |
| *Streptococcus* | Buccal swab | 0.192 | 0.017 | 0.084 |
| *Haemophilus* | Buccal swab | 0.168 | 0.027 | 0.084 |
| *Gemella* | Buccal swab | 0.060 | 0.201 | 0.402 |
| *Veillonella* | Buccal swab | 0.017 | 0.497 | 0.552 |
| *Fusobacterium* | Buccal swab | 0.030 | 0.366 | 0.523 |
| *Prevotella_7* | Buccal swab | 0.156 | 0.034 | 0.084 |
| *Neisseria* | Buccal swab | 0.186 | 0.020 | 0.084 |
| *Rothia* | Buccal swab | 0.040 | 0.296 | 0.493 |
| *Actinomyces* | Buccal swab | 0.021 | 0.458 | 0.552 |
| *Prevotella* | Buccal swab | 0.001 | 0.868 | 0.868 |
| *Prevotella_7* | Saliva | 0.009 | 0.632 | 0.776 |
| *Streptococcus* | Saliva | 0.061 | 0.198 | 0.659 |
| *Veillonella* | Saliva | 0.003 | 0.776 | 0.776 |
| *Haemophilus* | Saliva | 0.102 | 0.091 | 0.455 |
| *Fusobacterium* | Saliva | 0.005 | 0.726 | 0.776 |
| *Neisseria* | Saliva | 0.140 | 0.046 | 0.455 |
| *Prevotella* | Saliva | 0.018 | 0.490 | 0.763 |
| *Alloprevotella* | Saliva | 0.015 | 0.534 | 0.763 |
| *Campylobacter* | Saliva | 0.017 | 0.505 | 0.763 |
| *Gemella* | Saliva | 0.046 | 0.264 | 0.659 |
