# Supplementary material for: Effects of tobacco smoke and electronic cigarette vapor exposure on the oral and gut microbiota in humans: a pilot study
Source: PeerJ. 2018 Apr 30;6:e4693. doi: 10.7717/peerj.4693 (PMC5933315; doi:10.7717/peerj.4693)
Supplement: Supplemental Information 2 — Con, control; EC, electronic cigarette user, TS, tobacco smoker. [file peerj-06-4693-s002.docx]

**Table S2. Mean relative abundance of significant fecal taxa from the human cohort**

| Genera | Sample Type | Relative Abundance | | | |
| --- | --- | --- | --- | --- | --- |
|  |  | Overall Mean | Mean of Con | Mean of EC | Mean of TS |
| *Bacteroides* | Feces | 15.05 | 19.58 | 18.68 | 6.89 |
| *Bacteroides* | Buccal Swab | 0.07 | 0.06 | 0.06 | 0.08 |
| *Bacteroides* | Saliva | 0.07 | 0.02 | 0.02 | 0.17 |
| *Prevotella* | Feces | 10.68 | 2.72 | 0 | 29.33 |
| *Prevotella* | Buccal Swab | 0.01 | 0.01 | 0 | 0.02 |
| *Prevotella* | Saliva | 0.04 | 0 | 0.01 | 0.1 |

Con, control; EC, electronic cigarette user, TS, tobacco smoker
