# Supplementary figures and images for: Effects of tobacco smoke and electronic cigarette vapor exposure on the oral and gut microbiota in humans: a pilot study
Source: PeerJ. 2018 Apr 30;6:e4693. doi: 10.7717/peerj.4693 (PMC5933315; doi:10.7717/peerj.4693)

**A**

## Weighted UniFrac PCoA

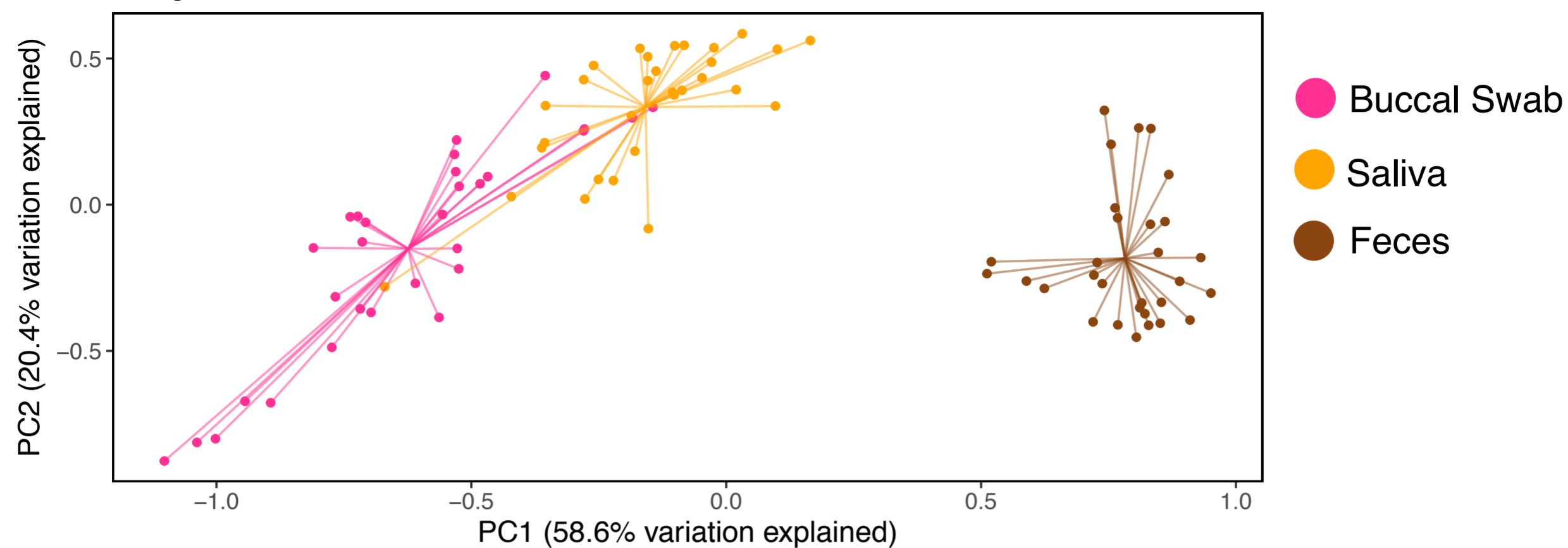**B**

Kruskal-Wallis P = 0.56

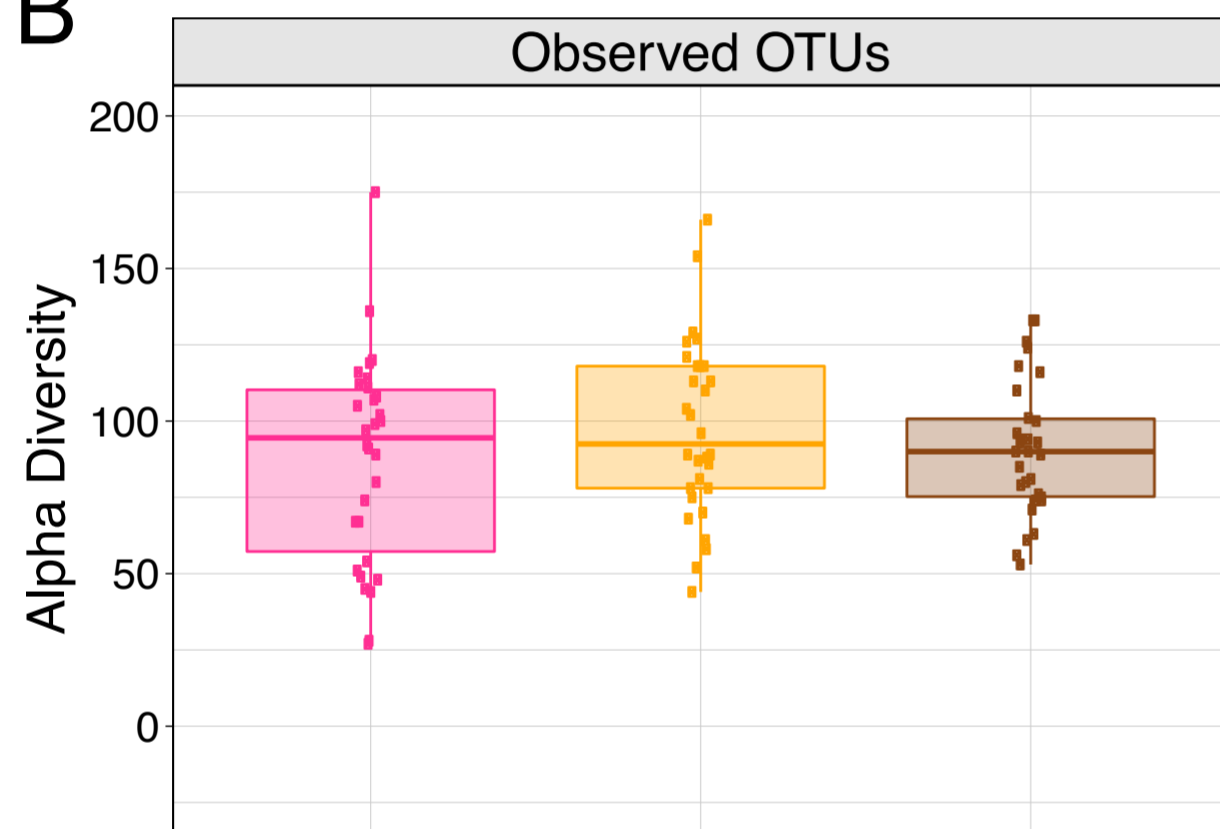

Kruskal-Wallis P &lt; 0.001

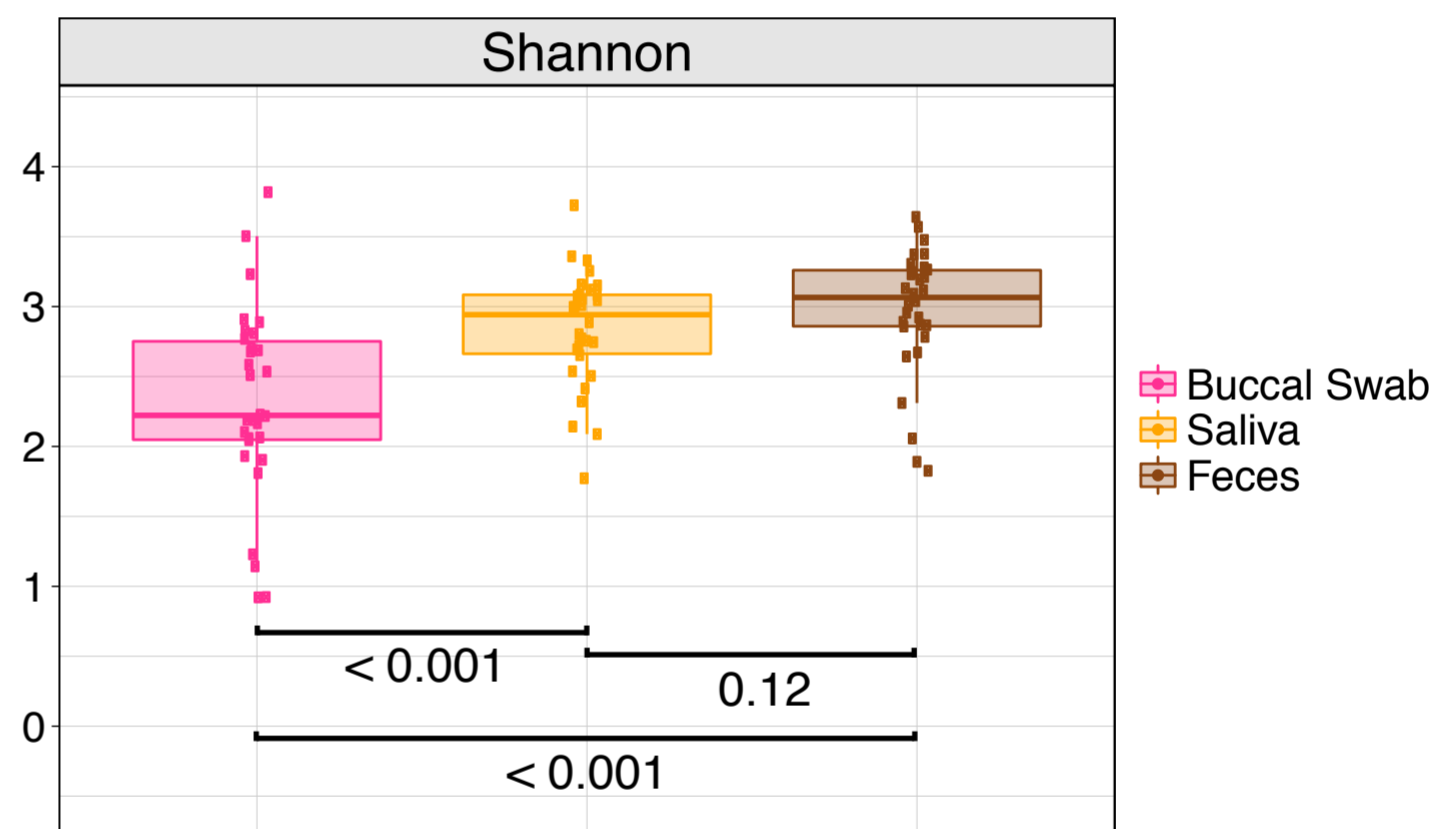**C**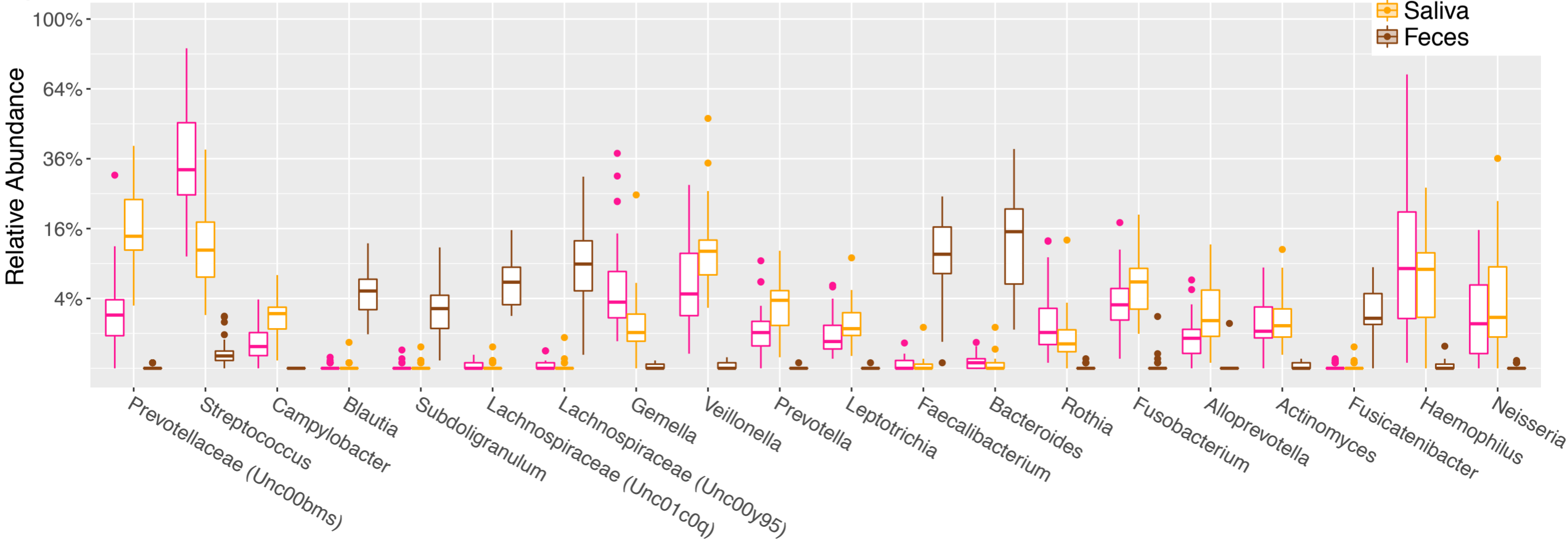

Supplement: Supplemental Information 3 — (A) Weighted UniFrac PCoA. (B) Alpha diversity. (C) Boxplot of most significant bacterial genera. All genera in the box plot were significantly different by Kruskal-Wallis with a P < 0.001. [file peerj-06-4693-s003.pdf]
