# Supplementary material for: Effects of tobacco smoke and electronic cigarette vapor exposure on the oral and gut microbiota in humans: a pilot study
Source: PeerJ. 2018 Apr 30;6:e4693. doi: 10.7717/peerj.4693 (PMC5933315; doi:10.7717/peerj.4693)
Supplement: Supplemental Information 4 — Genera ordered based on P value by reverse numerical order. All genera with >1% mean abundance included. No genera were found to be significantly different by exposure in either (A) Buccal swab or (B) Saliva. [file peerj-06-4693-s004.pdf]

A

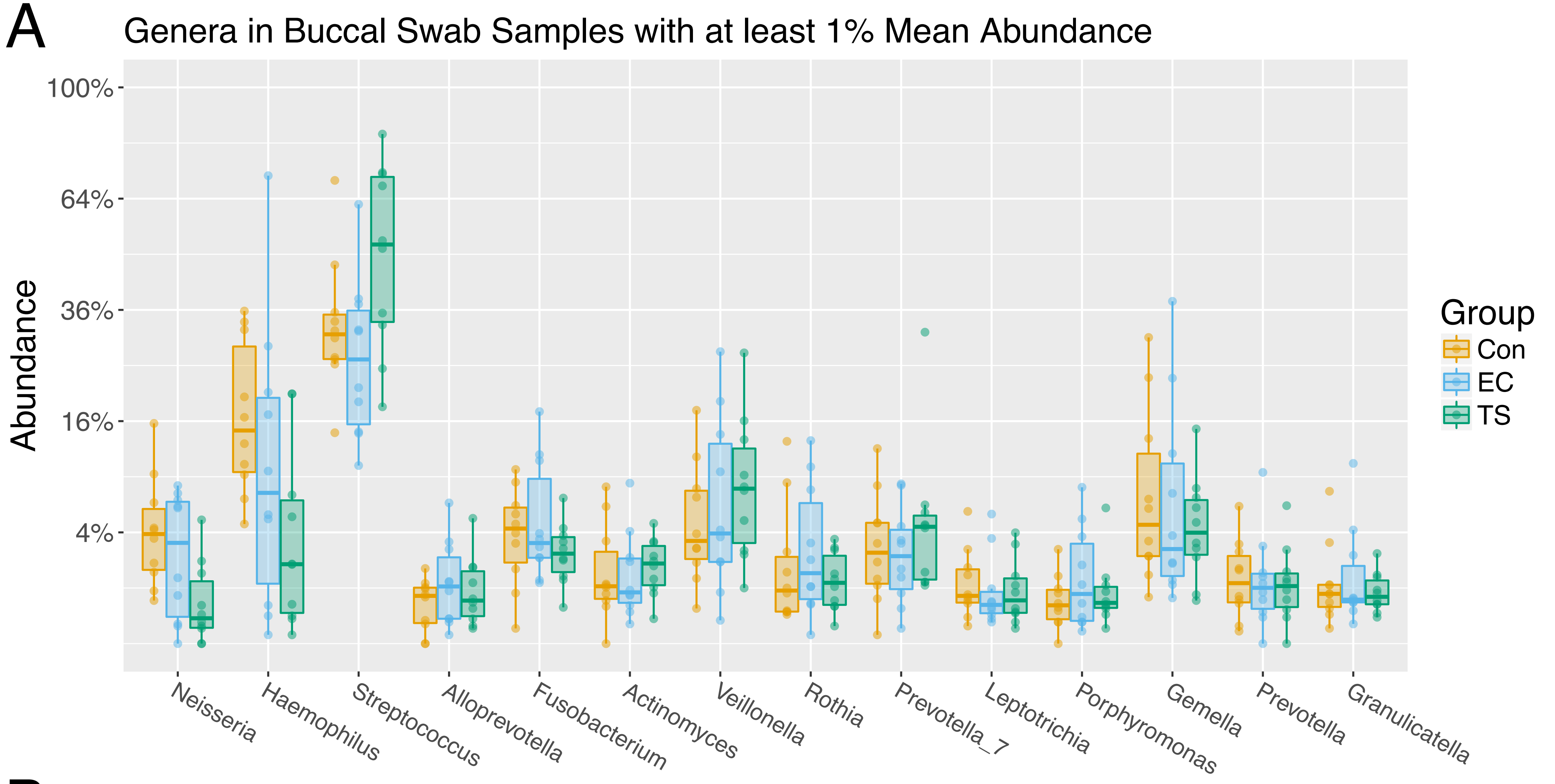
Genera in Buccal Swab Samples with at least 1% Mean Abundance
100%
64%
36%
16%
4%
Abundance
Neisseria
Haemophilus
Streptococcus
Alloprevotella
Fusobacterium
Actinomyces
Veillonella
Rothia
Prevotella_7
Leptotrichia
Porphyromonas
Gemella
Prevotella
Granulicatella
Group
Con
EC
TS

B

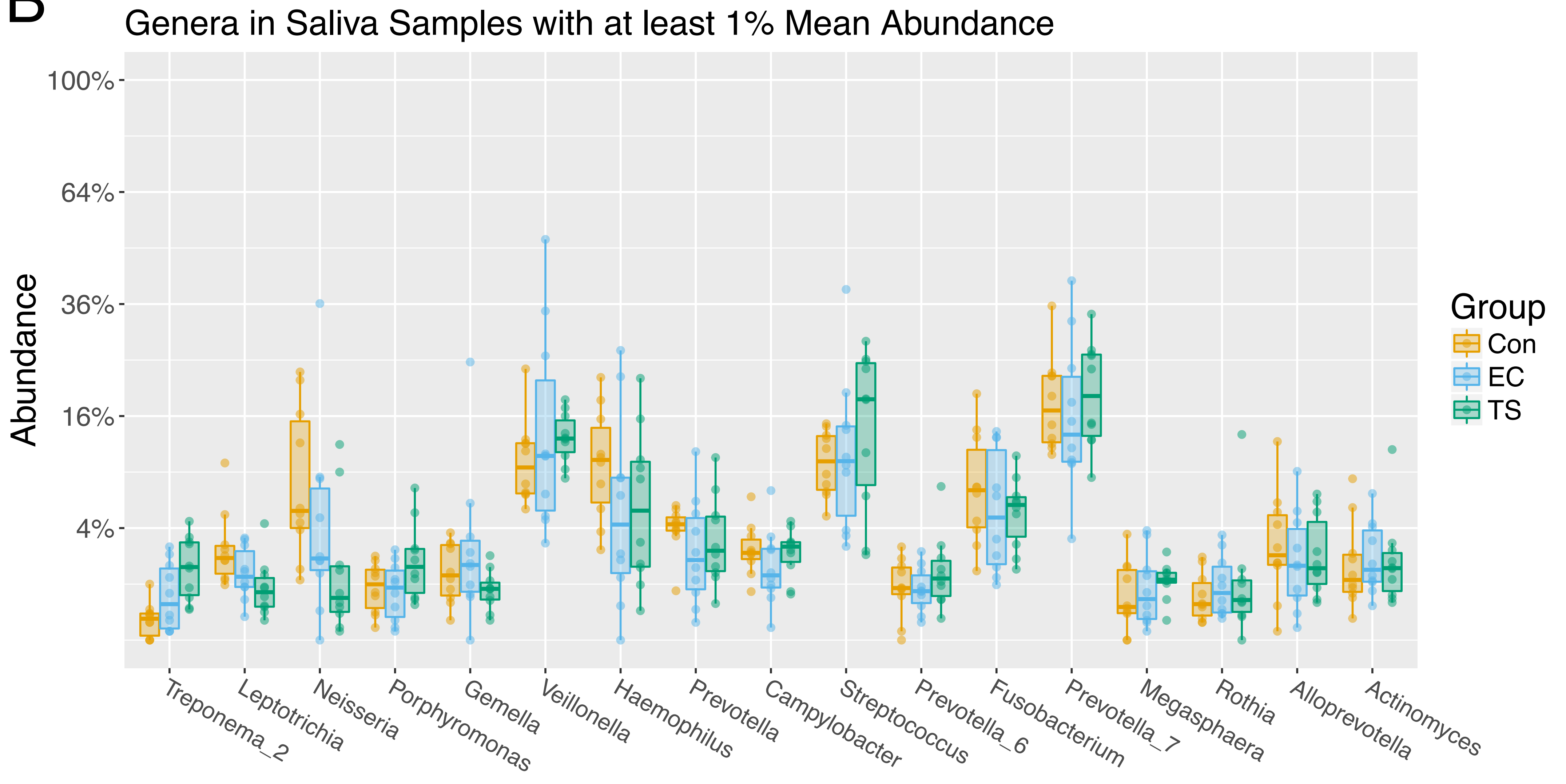
Genera in Saliva Samples with at least 1% Mean Abundance
100%
64%
36%
16%
4%
Abundance
Treponema_2
Leptotrichia
Neisseria
Porphyromonas
Gemella
Veillonella
Haemophilus
Prevotella
Campylobacter
Streptococcus
Prevotella_6
Fusobacterium
Prevotella_7
Megasphaera
Rothia
Alloprevotella
Actinomyces
Group
Con
EC
TS
