# Supplementary material for: Effects of tobacco smoke and electronic cigarette vapor exposure on the oral and gut microbiota in humans: a pilot study
Source: PeerJ. 2018 Apr 30;6:e4693. doi: 10.7717/peerj.4693 (PMC5933315; doi:10.7717/peerj.4693)
Supplement: Supplemental Information 5 — Correlations are based on linear regression. [file peerj-06-4693-s005.pdf]

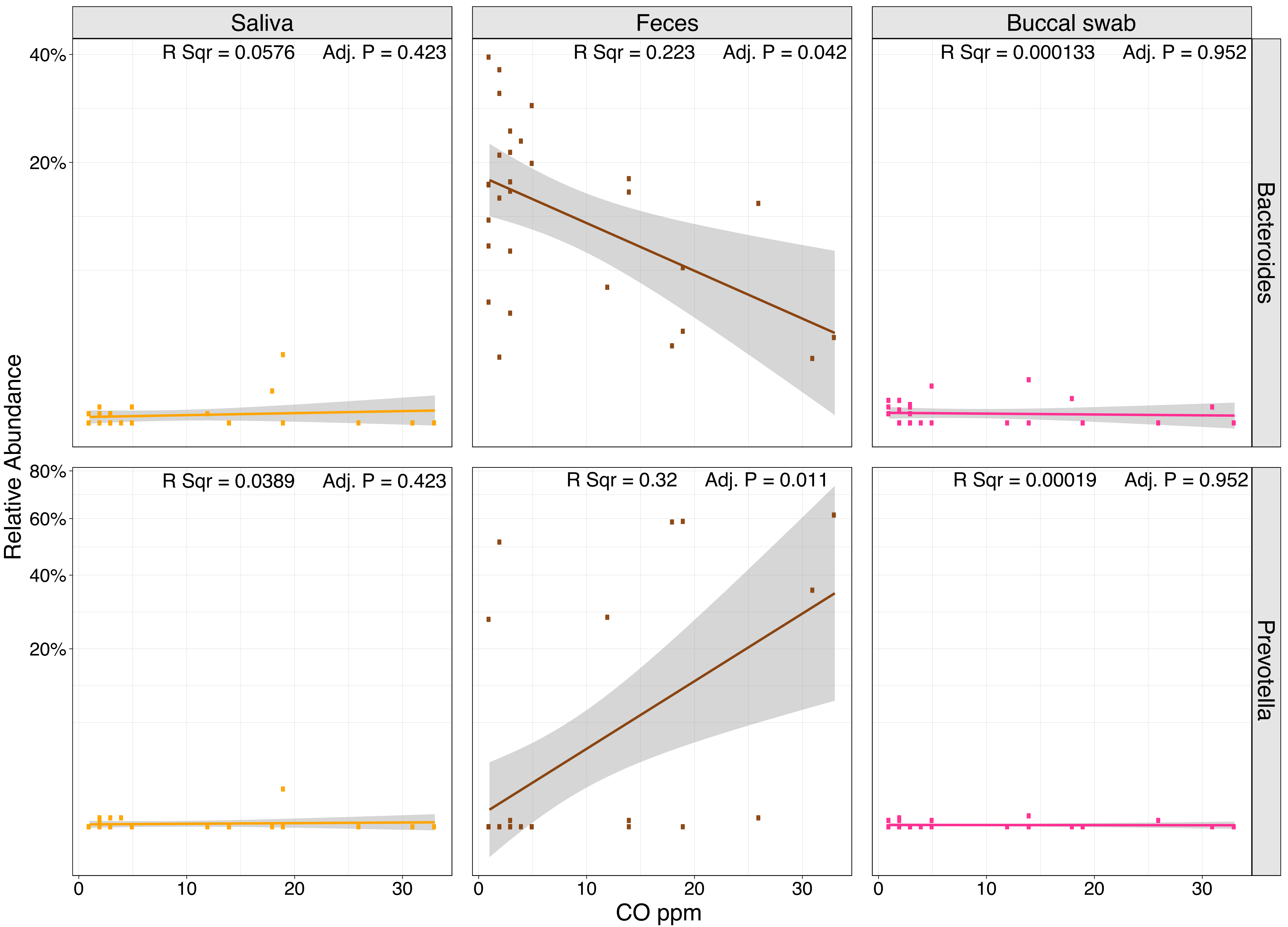

Saliva
Feces
Buccal swab
Bacteroides
Prevotella
R Sqr = 0.0576 Adj. P = 0.423
R Sqr = 0.223 Adj. P = 0.042
R Sqr = 0.000133 Adj. P = 0.952
R Sqr = 0.0389 Adj. P = 0.423
R Sqr = 0.32 Adj. P = 0.011
R Sqr = 0.00019 Adj. P = 0.952
Relative Abundance
40%
20%
80%
60%
40%
20%
0
10
20
30
CO ppm
