# Supplementary material for: Effects of tobacco smoke and electronic cigarette vapor exposure on the oral and gut microbiota in humans: a pilot study
Source: PeerJ. 2018 Apr 30;6:e4693. doi: 10.7717/peerj.4693 (PMC5933315; doi:10.7717/peerj.4693)
Supplement: Supplemental Information 8 [file peerj-06-4693-s008.html]

Ecig


# Ecig

#### *Daniel Smith*

#### *March 23, 2018*

## Data Import

```
suppressPackageStartupMessages({
  require(ape)
  require(DT)
  require(ggplot2)
  require(plyr)
  require(reshape2)
  require(rjson)
  require(slam)
  require(vegan)
})
```

```
## Warning: package 'ape' was built under R version 3.3.2
```

```
## Warning: package 'ggplot2' was built under R version 3.3.2
```

```
## Warning: package 'slam' was built under R version 3.3.2
```

```
## Warning: package 'vegan' was built under R version 3.3.2
```

```
metadata   <- read.delim("MetaData_Ecig_Final.txt")
json       <- rjson::fromJSON(file="Data_Ecig_Final.biom")
WeightedDM <- as.matrix(read.csv("Weighted_UniFrac_Ecig.csv", row.names = 1))

TaxaIDs   <- sapply(json$rows,    function (x) x$id)
SampleIDs <- sapply(json$columns, function (x) x$id)

# Parse the BIOM file (sparse abundance matrix encoded in JSON)
otus <- slam::simple_triplet_matrix(
          i        = sapply(json$data, function (x) x[[1]]) + 1,
          j        = sapply(json$data, function (x) x[[2]]) + 1,
          v        = sapply(json$data, function (x) x[[3]]),
          nrow     = length(TaxaIDs),
          ncol     = length(SampleIDs),
          dimnames = list(TaxaIDs, SampleIDs))

rownames(metadata)          <- metadata[['Sample']]
metadata                    <- metadata[colnames(otus),]
metadata[['Observed OTUs']] <- colapply_simple_triplet_matrix(otus, function (x) { sum(x>0) })
metadata[['Shannon']]       <- colapply_simple_triplet_matrix(otus, function (x) {
                                 x <- x[x>0]; -1 * sum((x / sum(x)) * log(x / sum(x))) })

# Roll up OTU abundances by genus and convert to relative abundance (%)
otu2genus <- setNames(
  nm     = sapply(json$rows, function (x) { x$id }),
  object = sapply(json$rows, function (x) {
    paste(collapse = "; ", sub("^_+", "", x$metadata$taxonomy))
  })
)
otu2genus <- factor(unname(otu2genus[rownames(otus)]))
genera    <- slam::rollup(otus, 1L, otu2genus, sum)
genera$v  <- genera$v / col_sums(genera)[genera$j]


remove("otus", "otu2genus", "json", "TaxaIDs", "SampleIDs")
```

## Figure 1

```
long <- melt(
  data          = metadata[,c("SampleType", "Group", "Observed OTUs", "Shannon")], 
  id.vars       = c("SampleType", "Group"), 
  variable.name = "Metric", 
  value.name    = "Diversity" )

for (i in levels(long[['SampleType']])) {
  df <- subset(long, SampleType==i)
  
  gg <- ggplot(df, aes(x=Group, y=Diversity, color=Group, fill=Group)) +
    geom_boxplot(position=position_dodge(.79), outlier.shape=NA, alpha=.3, size=1) +
    geom_point(position=position_dodge(.79), alpha=.5, size=1.8) +
    facet_wrap('Metric', scales = "free_y") + ylim(0, NA) + 
    scale_fill_manual( values = c("Con"="#e69f00", "EC"="#56b4e9", "TS"="#009e73")) + 
    scale_color_manual(values = c("Con"="#e69f00", "EC"="#56b4e9", "TS"="#009e73")) + 
    labs(x=NULL, y=i) + 
    theme(text = element_text(size=20), legend.position = 'none')
  print(gg)
}
```

```
stats <- ddply(long, c('SampleType', 'Metric'), function (x) {
  
  pVals <- sapply(
    X   = list(c("Con", "EC"), c("Con", "TS"), c("EC", "TS")), 
    FUN = function (y) {
      suppressWarnings({
        wilcox.test(
          x = x[x[['Group']] == y[1], 'Diversity'], 
          y = x[x[['Group']] == y[2], 'Diversity']
        )$p.value
      })
  })
  
  pVals <- c(pVals, kruskal.test(x[['Diversity']] ~ x[['Group']])$p.value)
  
  data.frame(
    'Comparison' = c("Con vs. EC", "Con vs. TS", "EC vs. TS", "All vs. All"), 
    'Statistic'  = c(rep("Mann-Whitney", 3), "Kruskal-Wallis"),
    'P-Value'    = signif(pVals, 3) )
})
datatable(stats, rownames = FALSE, class = "display compact")
```

```
remove("df", "long", "i", "gg", "stats")
```

## Figure 2

```
for (i in levels(metadata[['SampleType']])) {
  
  ids <- rownames(subset(metadata, SampleType == i))
  dm  <- as.dist(WeightedDM[ids, ids])
  res <- ape::pcoa(dm)
  ord <- data.frame(res$vectors[,1:2], Group=metadata[ids,'Group'])
  eig <- signif(res$values$Relative_eig[1:2] * 100, 3)
  
  df <- ddply(ord, 'Group', function (x) {
    x$cx <- mean(x[['Axis.1']])
    x$cy <- mean(x[['Axis.2']])
    return (x)
  })
  
  gg <- ggplot(df) +
    geom_point(aes(x=Axis.1, y=Axis.2, color=Group, fill=Group), size=2) +
    geom_segment(aes(x=Axis.1, y=Axis.2, color=Group, xend=cx, yend=cy), size=.75, alpha=0.4) +
    scale_color_manual(values = c("Con"="#e69f00", "EC"="#56b4e9", "TS"="#009e73")) +
    xlab(paste0("PC1 (", eig[1], "% variation explained)")) +
    ylab(paste0("PC2 (", eig[2], "% variation explained)")) +
    ggtitle(paste0(i, ", Weighted UniFrac PCoA")) + 
    theme(
      panel.border     = element_rect(color="black", fill=F, size=1),
      panel.grid.major = element_blank(), 
      panel.grid.minor = element_blank(),
      panel.background = element_rect(fill="white"),
      text             = element_text(size=14),
      plot.title       = element_text(size=16) )
  print(gg)
}
```

```
stats <- ddply(metadata, 'SampleType', function (x) {
  
  pVals <- sapply(
    X = list(c("Con", "EC"), c("Con", "TS"), c("EC", "TS"), c("Con", "EC", "TS")), 
    FUN = function (y) {
      
      ids <- as.character(subset(x, Group %in% y)[['Sample']])
      mtx <- WeightedDM[ids, ids]
      
      perms <- t(replicate(999, sample(1:nrow(mtx), nrow(mtx))))
      obj   <- vegan::adonis(mtx ~ metadata[rownames(mtx),'Group'], permutations=perms)
      
      return (obj$aov.tab[['Pr(>F)']][[1]])
  })
  
  data.frame(
    'Compairson' = c("Con vs. EC", "Con vs. TS", "EC vs. TS", "All vs. All"), 
    'Statistic'  = "Permanova",
    'P-Value'    = pVals )
})

datatable(stats, rownames = FALSE, class = "display compact")
```

```
remove("df", "gg", "ord", "res", "stats", "dm", "eig", "i", "ids")
```

## Figure 3

```
# Limit to only the Feces samples
md <- subset(metadata, SampleType == "Feces")
gn <- genera[,rownames(md)]

# Exclude taxa with less than 1% avg abundance
gn <- as.matrix(gn)[row_means(gn) >= .01,]
rownames(gn) <- sub("^.*; ", "", rownames(gn))

long <- melt(gn, varnames=c('Genus', 'Sample'), value.name="Abundance")
long[['Group']] <- md[as.character(long[['Sample']]), 'Group']

# How significant are the differences in abundance?
stats <- ddply(long, 'Genus', function (x) {
  data.frame(P.Value = signif(kruskal.test(x[['Abundance']] ~ x[['Group']])$p.value, 3))
})
stats[['FDR.Adj']] <- signif(p.adjust(stats[['P.Value']], method = 'fdr'), 3)

# Arrange Genera on plot from most to least significant
long[['Genus']]    <- factor(
  x      = as.character(long[['Genus']]), 
  levels = stats[['Genus']][order(stats[['P.Value']])] )

ggplot(long, aes(x=Genus, y=Abundance, color=Group, fill=Group)) +
  geom_boxplot(position=position_dodge(.79), outlier.shape=NA, alpha=.3) +
  geom_point(position=position_dodge(.79), alpha=.5) +
  scale_fill_manual( values = c("Con"="#e69f00", "EC"="#56b4e9", "TS"="#009e73")) +
  scale_color_manual(values = c("Con"="#e69f00", "EC"="#56b4e9", "TS"="#009e73")) +
  scale_y_sqrt(labels=scales::percent, limits=c(0,1), breaks=c(.04, .16, .36, .64, 1)) + 
  theme(
    text        = element_text(size=18),
    plot.title  = element_text(size=18),
    axis.text.x = element_text(angle = -30, vjust = 1, hjust = 0, size = 12) ) +
  labs(title="Genera in Feces Samples with at least 1% Mean Abundance", x=NULL)
```

```
datatable(stats, rownames = FALSE, class = "display compact")
```

```
remove("gn", "long", "md", "stats")
```

## Supp Figure 1

### S1 - A

```
dm  <- as.dist(WeightedDM)
res <- ape::pcoa(dm)
ord <- data.frame(res$vectors[,1:2], SampleType=metadata[rownames(res$vectors), 'SampleType'])
eig <- signif(res$values$Relative_eig[1:2] * 100, 3)

df <- ddply(ord, 'SampleType', function (x) {
  x$cx <- mean(x[['Axis.1']])
  x$cy <- mean(x[['Axis.2']])
  return (x)
})

ggplot(df) +
  geom_point(aes(x=Axis.1, y=Axis.2, color=SampleType)) +
  geom_segment(aes(x=Axis.1, y=Axis.2, color=SampleType, xend=cx, yend=cy), alpha=0.5) +
  xlab(paste0("PC1 (", eig[1], "% variation explained)")) +
  ylab(paste0("PC2 (", eig[2], "% variation explained)")) +
  ggtitle("Weighted UniFrac PCoA") + 
  theme(
    panel.border     = element_rect(color="black", fill=F, size=1),
    panel.grid.major = element_blank(), 
    panel.grid.minor = element_blank(),
    panel.background = element_rect(fill="white"),
    text             = element_text(size=14),
    plot.title       = element_text(size=16) )
```

```
remove("df", "ord", "res", "dm", "eig")
```

### S1 - B

```
long <- melt(
  data          = metadata[,c('SampleType', 'Observed OTUs', 'Shannon')], 
  id.vars       = "SampleType", 
  variable.name = "Metric", 
  value.name    = "Diversity" )

levels(long[['SampleType']]) <- sub("Buccal", "Buccal\n", levels(long[['SampleType']]))

ggplot(long, aes(x=SampleType, y=Diversity, color=SampleType, fill=SampleType)) +
  geom_boxplot(size=1, outlier.shape=NA, alpha=.3) +
  geom_point(alpha=.5) +
  facet_wrap('Metric', scales = "free_y") + 
  ylim(0, NA) + 
  xlab(NULL) + 
  theme(text = element_text(size=20), legend.position = 'none' )
```

```
remove("long")
```

### S1 - C

```
# Exclude taxa with less than 1% avg abundance
gn <- as.matrix(genera)[row_means(genera) >= .01,]
rownames(gn) <- sub("^.*; ", "", rownames(gn))

long <- melt(as.matrix(gn), varnames=c('Genus', 'Sample'), value.name="Abundance")
long[['SampleType']] <- metadata[as.character(long[['Sample']]), 'SampleType']

# How significant are the differences in abundance?
stats <- ddply(long, 'Genus', function (x) {
  data.frame(
    P.Value = signif(kruskal.test(x[['Abundance']] ~ x[['SampleType']])$p.value, 3),
    Feces   = signif(mean(subset(x, SampleType == "Feces")[['Abundance']]), 3),
    Saliva  = signif(mean(subset(x, SampleType == "Saliva")[['Abundance']]), 3),
    Swab    = signif(mean(subset(x, SampleType == "Buccal Swab")[['Abundance']]), 3))
})
stats[['FDR.Adj']] <- p.adjust(stats[['P.Value']], method = 'fdr')
stats <- stats[order(stats[['P.Value']]),]

# Limit to top 20, order from most to least significant
top20 <- as.character(head(stats[['Genus']], 20))
long  <- long[long[['Genus']] %in% top20,]
long[['Genus']] <- sub("^.+; ([A-Z])", "\\1", as.character(long[['Genus']]))
long[['Genus']] <- factor(long[['Genus']], sub("^.+; ([A-Z])", "\\1", top20))

ggplot(long, aes(x=Genus, y=Abundance, color=SampleType, fill=SampleType)) +
  geom_boxplot(position=position_dodge(.79), outlier.shape=NA, alpha=.3) +
  geom_point(position=position_dodge(.79), alpha=.5) +
  scale_y_sqrt(labels=scales::percent, limits=c(0,1.1), breaks=c(.04, .16, .36, .64, 1)) + 
  theme(
    text        = element_text(size=15),
    plot.title  = element_text(size=15),
    axis.text.x = element_text(angle = -30, vjust = 1, hjust = 0, size = 10),
    plot.margin = unit(x=c(.01, .08, .01, .01), units="npc"),
    legend.position = 'bottom' ) +
  ggtitle("Top 20 Most Significant Differences in Genera with at least 1% Mean Abundance") +
  xlab(NULL)
```

```
datatable(stats, rownames = FALSE, class = "display compact")
```

```
remove("gn", "long", "top20", "stats")
```

## Supp Figure 2

### S2 - A

```
# Limit to only the Buccal Swab samples
md <- subset(metadata, SampleType == "Buccal Swab")
gn <- genera[,rownames(md)]

# Exclude taxa with less than 1% avg abundance
gn <- as.matrix(gn)[row_means(gn) >= .01,]
rownames(gn) <- sub("^.*; ", "", rownames(gn))

long <- melt(gn, varnames=c('Genus', 'Sample'), value.name="Abundance")
long[['Group']] <- md[as.character(long[['Sample']]), 'Group']

# How significant are the differences in abundance?
stats <- ddply(long, 'Genus', function (x) {
  data.frame(
    P.Value = signif(kruskal.test(x[['Abundance']] ~ x[['Group']])$p.value, 3),
    Con     = signif(mean(subset(x, Group == "Con")[['Abundance']]), 3),
    EC      = signif(mean(subset(x, Group == "EC")[['Abundance']]), 3),
    TS      = signif(mean(subset(x, Group == "TS")[['Abundance']]), 3)
  )
})
stats[['FDR.Adj']] <- signif(p.adjust(stats[['P.Value']], method = 'fdr'), 3)

# Arrange Genera on plot from most to least significant
long[['Genus']]    <- factor(
  x      = as.character(long[['Genus']]), 
  levels = stats[['Genus']][order(stats[['P.Value']])] )

ggplot(long, aes(x=Genus, y=Abundance, color=Group, fill=Group)) +
  geom_boxplot(position=position_dodge(.79), outlier.shape=NA, alpha=.3) +
  geom_point(position=position_dodge(.79), alpha=.5) +
  scale_fill_manual( values = c("Con"="#e69f00", "EC"="#56b4e9", "TS"="#009e73")) +
  scale_color_manual(values = c("Con"="#e69f00", "EC"="#56b4e9", "TS"="#009e73")) +
  scale_y_sqrt(labels=scales::percent, limits=c(0,1), breaks=c(.04, .16, .36, .64, 1)) + 
  theme(
    text        = element_text(size=18),
    plot.title  = element_text(size=18),
    axis.text.x = element_text(angle = -30, vjust = 1, hjust = 0, size = 12) ) +
  xlab(NULL) + ggtitle("Genera in Buccal Swab Samples with at least 1% Mean Abundance")
```

```
datatable(stats, rownames = FALSE, class = "display compact")
```

```
remove("gn", "long", "md", "stats")
```

### S2 - B

```
# Limit to only the Saliva samples
md <- subset(metadata, SampleType == "Saliva")
gn <- genera[,rownames(md)]

# Exclude taxa with less than 1% avg abundance
gn <- as.matrix(gn)[row_means(gn) >= .01,]
rownames(gn) <- sub("^.*; ", "", rownames(gn))

long <- melt(gn, varnames=c('Genus', 'Sample'), value.name="Abundance")
long[['Group']] <- md[as.character(long[['Sample']]), 'Group']

# How significant are the differences in abundance?
stats <- ddply(long, 'Genus', function (x) {
  data.frame(
    P.Value = signif(kruskal.test(x[['Abundance']] ~ x[['Group']])$p.value, 3),
    Con     = signif(mean(subset(x, Group == "Con")[['Abundance']]), 3),
    EC      = signif(mean(subset(x, Group == "EC")[['Abundance']]), 3),
    TS      = signif(mean(subset(x, Group == "TS")[['Abundance']]), 3)
  )
})
stats[['FDR.Adj']] <- signif(p.adjust(stats[['P.Value']], method = 'fdr'), 3)

# Arrange Genera on plot from most to least significant
long[['Genus']]    <- factor(
  x      = as.character(long[['Genus']]), 
  levels = stats[['Genus']][order(stats[['P.Value']])] )

ggplot(long, aes(x=Genus, y=Abundance, color=Group, fill=Group)) +
  geom_boxplot(position=position_dodge(.79), outlier.shape=NA, alpha=.3) +
  geom_point(position=position_dodge(.79), alpha=.5) +
  scale_fill_manual( values = c("Con"="#e69f00", "EC"="#56b4e9", "TS"="#009e73")) +
  scale_color_manual(values = c("Con"="#e69f00", "EC"="#56b4e9", "TS"="#009e73")) +
  scale_y_sqrt(labels=scales::percent, limits=c(0,1), breaks=c(.04, .16, .36, .64, 1)) + 
  theme(
    text        = element_text(size=18),
    plot.title  = element_text(size=18),
    axis.text.x = element_text(angle = -30, vjust = 1, hjust = 0, size = 12) ) +
  labs(x=NULL, title="Genera in Saliva Samples with at least 1% Mean Abundance")
```

```
datatable(stats, rownames = FALSE, class = "display compact")
```

```
remove("gn", "long", "md", "stats")
```

## S3

```
stats <- ddply(metadata, 'SampleType', function (md) {
  
  rownames(md) <- as.character(md[['Sample']])
  
  # Find the top 10 most abundant genera with minimum avg abundance >= 1%
  gn    <- genera[,rownames(md)]
  gn    <- gn[row_means(gn) >= .01,]
  top10 <- head(names(rev(sort(row_means(genera)))), 10)
  gn    <- as.matrix(genera[top10,rownames(md)])
  
  # Compute correlation between each genus and CO ppm
  long <- melt(gn, varnames=c('Genus', 'Sample'), value.name="Abundance")
  long[['CO_ppm']] <- md[as.character(long[['Sample']]), 'CO_ppm']
  
  result <- ddply(long, 'Genus', function (x) {
    obj <- lm(Abundance ~ CO_ppm, data=x)
    data.frame(
      'R.Squared' = signif(summary(obj)$r.squared, 3),
      'P.Value'   = signif(coef(summary(obj))['CO_ppm', 'Pr(>|t|)'], 3)
      )
  })
  result[['FDR.Adj']] <- signif(p.adjust(result[['P.Value']], method = 'fdr'), 3)
  
  return (result)
})
signifTaxa <- as.character(stats[['Genus']][stats[['FDR.Adj']] <= 0.05])
stats[['Genus']] <- sub("^.+; ([A-Z])", "\\1", as.character(stats[['Genus']]))

# Plot correlations for significantly correlated genera
long <- melt(
  data       = as.matrix(genera[signifTaxa,]),
  varnames   = c("Genus", "Sample"),
  value.name = "Abundance"
)
long[['SampleType']] <- metadata[as.character(long[['Sample']]), 'SampleType']
long[['CO_ppm']]     <- metadata[as.character(long[['Sample']]), 'CO_ppm']
long[['Genus']]      <- sub("^.+; ([A-Z])", "\\1", as.character(long[['Genus']]))

ggplot(long, aes(x=CO_ppm, y=Abundance, color=SampleType, fill=SampleType)) +
  geom_point() + 
  geom_smooth(method = "lm") +
  facet_grid(Genus ~ SampleType) +
  scale_y_continuous(labels=scales::percent) + 
  theme(
    text            = element_text(size=18),
    plot.title      = element_text(size=18),
    legend.position = "none" ) +
  ggtitle("Genera Correlated with CO ppm in Fecal Samples")
```

```
datatable(stats, rownames = FALSE, class = "display compact")
```

```
remove("long", "signifTaxa", "stats")
```
